# Non-Hodgkin’s Lymphoma as a Risk Factor for Persistent Chylothorax After Transhiatal Esophagectomy

**DOI:** 10.4021/wjon523w

**Published:** 2012-10-28

**Authors:** Casey J. Allen, Peter J. DiPasco, Vadim Koshenkov, Dido Franceschi

**Affiliations:** a Dewitt Daughtry Family Department of Surgery, Division of Surgical Oncology, University of Miami Miller School of Medicine, Clinical Research Building ,4th Floor (C232), 1120 NW 14th Street, Miami, FL, 33136, USA

**Keywords:** Transhiatal esophagectomy, Chylothorax, Lymphoma, Thoracic duct, Esophageal adenocarcinoma

## Abstract

We report a case of an 82 years old female with Non-Hodgkin Lymphoma (NHL) in remission whom underwent a transhiatal esophagectomy (THE) for esophageal adenocarcinoma. The post-operative course was complicated by severe chylothorax requiring an additional thoracotomy for ligation of the thoracic duct. The influence of the patient’s history of NHL on the development of such a severe chylothorax is under question.

## Introduction

Despite refinements of operative techniques employed in transhiatal esophagectomy, injury to the thoracic duct remains a beleaguering problem with significant attendant post-operative morbidity. Non-surgical (spontaneous) chylothorax can have several origins, but lymphoma, and particularly that of NHL, represents the most common etiology [1]. Herein we describe an additive situation of patient history and surgical injury resulting in severe prolonged chylothorax after transhiatal esophagectomy.

## Case Report

The index patient is an 82 years old female with a history of an intermediate grade malignant lymphoma, follicular type, in remission. The patient underwent combination chemotherapy with Cytotoxan, Novantrone, and Vincristine fourteen years prior. The patient remained free of recurrence in subsequent follow-up. Past medical history was also significant for hypertension, hypercholesterolemia, and a 90 pack-year tobacco use. Clinical workup revealed poorly-differentiated adenocarcinoma of the gastroesophageal junction pT3N2M0 after EUS and PET/CT assessment [2]. The patient underwent six cycles of neoadjuvant chemotherapy (NAC) consisting of Carboplatin and Paclitaxel. Satisfactory clinical response to NAC was achieved and resection was planned.

**Figure 1:**
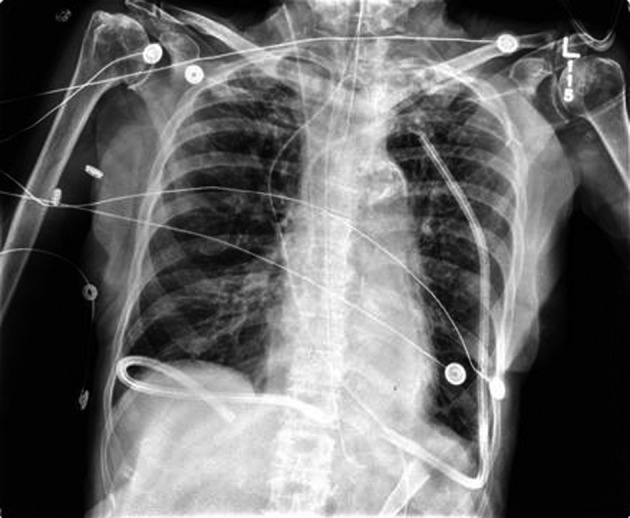
Immediate Post-operative Chest X-Ray following THE.

**Figure 2:**
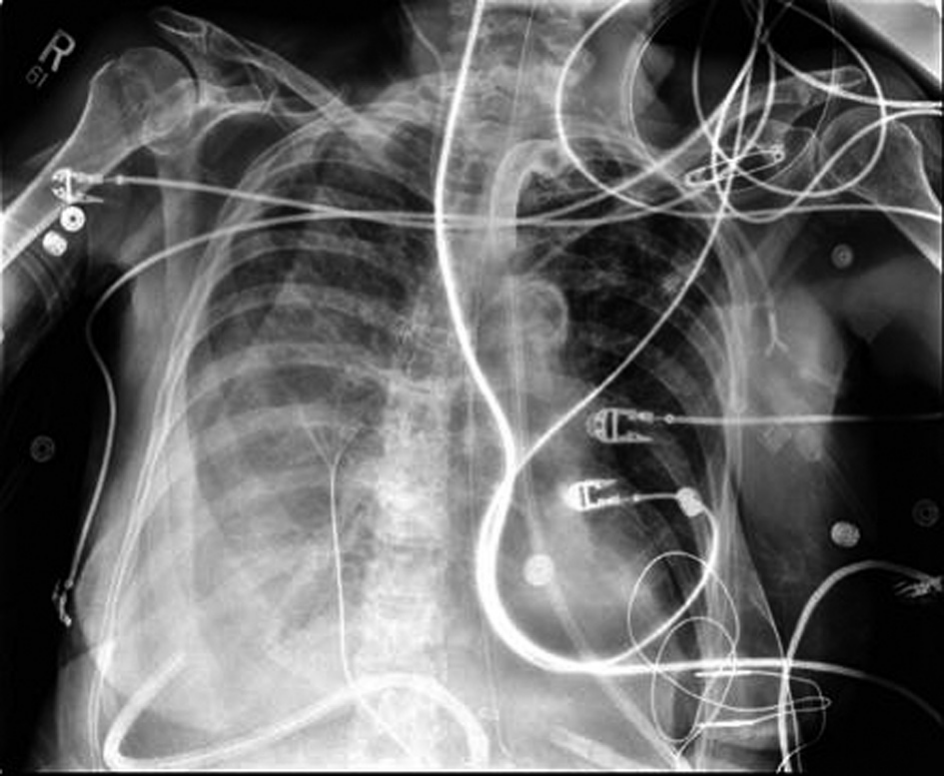
Post-operative day 9; development of right chylothorax.

The patient underwent a THE as well as placement of a feeding jejunostomy tube. As per our standard practice, Jackson-Pratt (JP) drains were placed in the pleural spaces bilaterally via the mediastinal hiatus to prevent the collection of any possible post-operative effusions (Fig. 1). The patient tolerated the procedure well but post-operatively the patient deteriorated into respiratory failure requiring re-intubation and transient vasopressor support. By the third post-operative day, a second attempt of extubation had failed and the decision for early tracheostomy placement was made to assist with long-term ventilatory support. Slightly more than one week post-operative, the right thoracic JP drain was noted to be draining about 5 L of serous fluid daily. There was evidence of increasing right sided pleural effusions on chest X-ray (Fig. 2). Drainage continued at an average of more than 4 liters per day, with a peak output of nearly 6 liters at one measurement. As enteral feeds were initiated via the jejunostomy, it was noted the pleural effluent changed from serous to white, opaque, and fatty. The suspicion of chyle leak was confirmed with elevated triglyceride level from the draining fluid. As enteral feeds were stopped, JP output moderately decreased and again became transparent. Octreotide and parenteral nutrition were initiated for attempted conservative management of the chylothorax. This management failed, however, as the daily chylous thoracic output persisted at an average of more than 3 L per day. A lymphoscintigram failed to locate the specific source of the lymphatic leak therefore targeted embolization/ligation of the thoracic duct via interventional radiology was precluded.

Considering the failure of nearly one month of conservative management, on post-operative day 36 the patient underwent a right thoracotomy with ligation of the thoracic duct. Enteral infusion of 300 mL of heavy cream assisted in localizing the injured thoracic duct at the time of operation. The leaking thoracic duct was mass ligated with non-absorbable suture and closure was assisted with the placement of Surgicel and Fibrin Glue.

**Figure 3:**
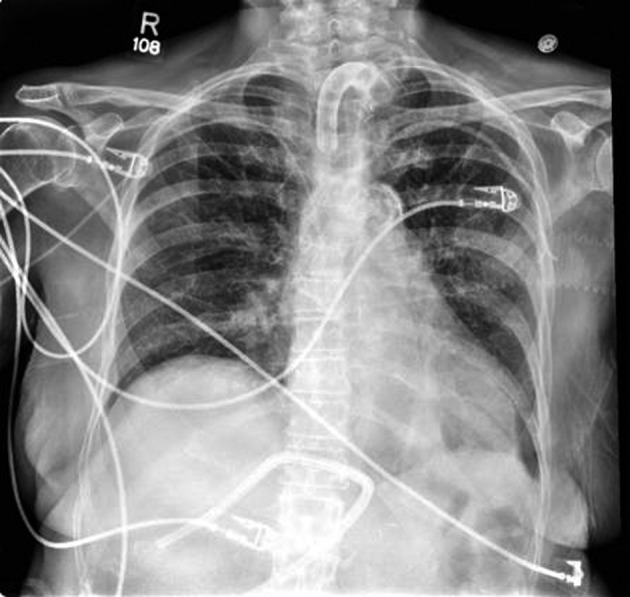
Chest X-Ray following ligation of thoracic duct and removal of drains, POD12.

After surgery, the thoracic drain output dropped significantly. At the time of discharge, the output averaged 200 mL per day. The patient was eventually taken off TPN and switched to tube feeds. The patient was also weaned from the ventilator and was tolerating 24 hour tracheostomy collar. All intrathoracic drains were removed (Fig. 3). The patient was discharged to a rehabilitation facility where the patient was able to be de-cannulated and receive the physical therapy required.

## Discussion

In an average adult, about 2.4 L of total chyle is transported through the lymphatic system every day [1]. Our 49 kg elderly female patient ranged between 3 - 5 L of chylous output from the thoracic drain per day. Not only is the incidence of post-operative chylothorax as a result of THE rare (1-4%), even more rare in the case of our patient is the voluminous output of chyle from this type of injury [3, 4].

Lymphoma, particularly NHL, is the number one cause of all chylothoraces [1]. To our knowledge, there is no literature that discusses the incidence of chylothorax after THE on patients with lymphoma. The incidence and cause of disruption of either scenario alone, however, is well understood.

In a recent large cohort study from the University of Michigan, over 2,000 transhiatal esophagectomies performed over 30 years showed the incidence of chylothorax was less than 1% [4]. Another recent, but smaller cohort from Germany showed that of 409 esphagectomies performed between 1988 and 2005, only 10 were complicated by post-operative chylothorax, and only 1 of those was performed through the transhiatal approach [5]. The average amount of post-operative chylous production from this study was 2.2 L per day [5].

As malignancy in general accounts for over half of all cases of chylothorax, the association between lymphoma and the development of a chylothorax is also well known [1, 6]. Many different types of cancers have been reported to cause chylothorax, with lymphoma being the most common, and non-Hodgkin's lymphoma being more likely than Hodgkin’s Lymphoma to cause chylothorax [1, 3-6]. Although it is not completely understood, malignancies are believed to cause chylothorax through compression of the thoracic duct from mediastinal adenopathy or mass effect. This leads to distention of the thoracic duct and its tributaries, making the lymphatic system vulnerable to rupture [6]. Other explanations have been theorized as chylothorax has been shown to develop in patients without evidence of distal lymphatic compression [7]. Another theory is that the large amount of lymphocytes and proteinacious material within the chyle associated with leukemia and lymphoma will cause a highly viscous lymphatic system, contributing to the high pressure on the wall of the thoracic duct. This high intraluminal pressure would contribute to the distention and vulnerability of the lymphatic duct system even further [7]. Any minor trauma, such as a deep cough or violent sneeze, may result in microdisruptions of this highly vulnerable luminal wall, thus causing chyle to drain into the pleural space.

Chylothorax itself is a severe complication. Chyle is composed of numerous substances including fat, cholesterol, vitamins, electrolytes, as well as lymphocytes, immunoglubulins and digestive enzymes [1]. The loss of these substances can result in numerous nutritional, metabolic and immunologic disorders that can be life threatening [1, 5]. Without treatment mortality can rise to over 50% [5, 8]. Current treatment modalities include conservative therapy consisting of total parenteral nutrition, octreotide, and percutaneous drainage [8]. Surgical therapy is generally reserved for those cases that do not resolve with conservative treatment, have high output (> 1 L per day), or develop morbid complications [8].

Although there is not much literature detailing the clinical intricacies of chylothorax associated with NHL, there are however several cases reports that describe its nature. One report detailed how a chylothorax developed in association with active NHL and ultimately requiring surgical intervention [9]. Another report described the use of conservative therapy as sufficient means to resolve the complication [10].

Chylothorax has even been reported in a patient with history of lymphoma in 9 years remission [11]. The authors theorize that the fragility of the thoracic duct increases, resulting in an increase risk of lymphatic leak, even in stable remission [11].

Interestingly enough, another report describes a case where a patient with NHL in remission presented with a retrosternal goiter and chylothorax, leading to the question of whether the history of lymphoma predisposed the patient to this complication [12].

There are clearly many causes of chylothorax, but it appears there may be certain situations that can predispose a patient to this complication. Our patient was in remission, but the effects of lymphoma may have increased the risk of iatrogenic rupture of the thoracic duct. There have been reports of chylothorax in patients in remission, as well has patients in remission with a coinciding disease process triggering a thoracic duct leak. Those with lymphoma seem to be at higher risk of chylothorax, even when in stable remission. Whether the lymphatics remain vulnerable due to residual systemic hypertrophy or there remain changes in the lymphatic fluid dynamics even in remission are all still speculative.

To our knowledge, we are the first to report a case of profound post-operative chylothorax in a patient with NHL in remission. We suggest there is an association that may have not only predisposed our patient to this complication, but may have also predisposed its severity.
